# The influence of adolescents’ self-perception of social relationships on personality functioning in the context of inclusive education

**DOI:** 10.3389/fpsyg.2024.1279623

**Published:** 2024-08-01

**Authors:** Anne Hartmann, Michel Knigge, Jenny Lenkeit, Antje Ehlert, Kirstin Goth, Nadine Spörer

**Affiliations:** ^1^Psychological Primary School Pedagogy, Structural Unit Educational Sciences, University of Potsdam, Potsdam, Germany; ^2^Inclusive School Development, Structural Unit Educational Sciences, University of Potsdam, Potsdam, Germany; ^3^Special Educational Needs with Focus Learning, Structural Unit Educational Science, University of Potsdam, Potsdam, Germany; ^4^Department of Child and Adolescent Psychiatry, University Clinics Saarland, Homburg, Germany

**Keywords:** social relationships, personality functioning, self-perception, adolescents, special educational needs, inclusion

## Abstract

**Introduction:**

Adolescence is a special phase of life in which fundamentals of psychosocial functioning are formed. The present study investigates how adolescents’ self-perception of social relationships in inclusive classes affect personality functioning. Furthermore, we examine whether the association between self-perceived social relationships and personality functioning is stronger for students with special educational needs in the domains of learning (SEN L) and social -emotional development (SED) than for students without SEN.

**Methods:**

At two measurement points questionnaire data was collected from 927 sixth- and seventh-graders from 20 primary and 20 comprehensive inclusive classes in Germany.

**Results:**

Results of longitudinal multilevel analyses show partially different results for sixth- and seventh-graders. Overall, students’ perceived social relatedness predicted personality functioning. Students who perceived their social relationships more positively showed healthier personality functioning. Further, SEN SED represents a potential risk factor for personality functioning. But, we observed that differences in personality functioning between seventh-graders with SEN L or SED and those without SEN decreased over time. Furthermore, SEN does not appear to reinforce the association between low self-perception of social relationships and risky personality functioning.

**Discussion:**

The findings are discussed in the context of inclusive education and implications for future research as well as practice are given. Overall, our findings contribute to a better understanding of students’ psychosocial development in inclusive learning environments.

## 1 Introduction

Adolescence is a special phase of life in which complex developmental tasks, such as forming relationships with peers or personality development, have to be mastered (König et al., 2011; Gniewosz and Titzmann, 2018). In adolescence, the complexity of social interactions increases and belonging to a peer group becomes increasingly important (Bossaert et al., 2013). Since adolescents spend almost one-third of their time at school, it has an important influence on their development of close social relationships and personality functioning through interaction with their peers (König et al., 2011; Rueger et al., 2016). According to Neyer and Asendorpf (2018), continuous interactions exist between personality and social relationships. Both have a significant influence on the successful accomplishment of developmental tasks as well as on adolescents’ mental health (Gniewosz and Titzmann, 2018). Therefore, it is important to take a closer look at both constructs and their dynamic development during adolescence. Further, meaningful social relationships with peers are discussed as a key issue for successful inclusive educational settings because students with special educational needs (SEN) are often reported to experience difficulties in participating fully in regular education settings (Bossaert et al., 2013; OECD, 2017). Studies involving adolescents with SEN, especially in the area of social–emotional development (SED), found that they feel less socially integrated in the social processes of their class compared to their peers without SEN (Koster et al., 2009; Schwab, 2015; Hartmann et al., 2022). Hence, it is essential to investigate the association between social relationships and personality development in inclusive classrooms. In our study, we focus on students with SEN learning (L) and SED, as these special needs are the most prevalent in Germany [KMK (Conference of the Ministers of Education and Cultural Affairs), 2022]. Students with SEN L are characterized by showing patterns of thinking and learning that can lead to disorientation when encountering and dealing with school learning objects. Students with SEN SED are characterized by difficulties in emotional and social development, cooperation, attention, and self-regulation (*ibid*). In contrast to the majority of previous research on this topic, in the present study, we investigate the association between self-perceived social relationships and personality functioning from the perspective of adolescents themselves. Furthermore, we examine whether students with SEN L and SED have different developmental trajectories of personality functions compared to students without SEN. Finally, we investigate whether SEN L and SED reinforces the influence of students’ self-perception of their social relationships on personality functioning.

## 2 Inclusive education

In 2009, Germany ratified the UN Convention and committed to enable students with different social backgrounds, intellectual abilities, and developmental requirements to participate in mainstream classrooms as comprehensively as possible. The Standing Conference of the Ministers of Education and Cultural Affairs in Germany (KMK) has determined SEN in the domains of learning, social–emotional, language, sight, hearing, intellectual, and physical and motor skills development. In 2020, around 582,400 students with SEN were taught in Germany of which almost 255,000 attended inclusive mainstream schools. Of the total SEN, 57% was allocated to the domain of L and SED [KMK (Conference of the Ministers of Education and Cultural Affairs), 2022]. The shift to inclusive education aimed to forgo assessment procedures in order to avoid labelling or stigmatization, and to provide additional support for all students who need it (Vroey et al., 2016). However, in Germany, the allocation of staff and finances is partly linked to the number of students formally identified with SEN. Thus, the diagnostic process is not yet completely abandoned [KMK (Conference of the Ministers of Education and Cultural Affairs), 2022], as for example in the USA or Norway (Bossaert et al., 2015). For the schools investigated, where students with and without SEN learned together in one and the same classroom, psychometric diagnoses were not mandatory.

## 3 Theoretical and empirical background

**Figure 1:**
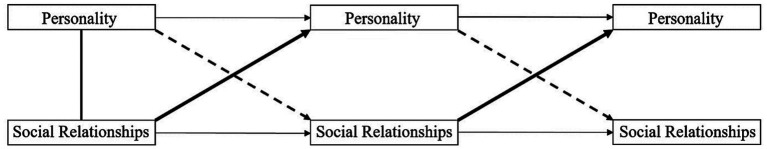
Dynamic personality-relationship-transaction model (Neyer and Asendorpf, 2018).

According to Asendorpf (2019), a persons’ personality is generally understood as the totality of all personality traits. In the course of personality development, there are dynamic interactions between personality and genetic influences on the one hand and personality and environmental influences on the other hand (Asendorpf, 2019). Thus, personality development is sensitive to social environmental influences (Asendorpf et al., 2017). Social relationships, such as those of an adolescent to his or her peers, can be considered a special form of the social environment (Neyer et al., 2014; Neyer and Asendorpf, 2018). According to the dynamic-interactionist paradigm, continuous interactions exist between social relationships and personality (Neyer and Asendorpf, 2018). Such special interactions over time are also known as dynamic personality-relationship-transactions (see Figure 1). Dynamic transactions lead to a fit between personality and social relationships (Asendorpf et al., 2017).

Neyer and Asendorpf (2018) argue that interactions tend to occur in the context of life transitions that are often tied to a specific age. In the context of non-normative transitions, which are less regulated by social expectations, personality has a stronger influence on relationship characteristics (Neyer et al., 2014; Neyer and Asendorpf, 2018). In contrast, normative transitions may be, e.g., adolescence as a transition from childhood to adulthood or the transition from primary to secondary school. In such normative transitions a strong influence of social relationships on personality is postulated. Mastering and adapting to rather normative life transitions depends much more on characteristics of the social relationships than on personality. Thus, age-related discontinuities have the potential to catalyze personality change because they exert strong pressures and provide clear advice for adaptive behavior (Neyer et al., 2014). Normative transitions can set personality change in motion, while non-normative transitions can paradoxically be ambiguous and unpredictable and, therefore, better managed by the personality a person holds (Neyer et al., 2014).

Several studies investigating personality-relationship transactions (e.g., Neyer and Asendorpf, 2001; Neyer and Lehnart, 2007; Wagner et al., 2015) found that changes in meaningful relationships in young adulthood, such as the first partnership, have the potential to influence personality and its development. High quality relationships lead to higher self-esteem, conscientiousness, extraversion, and a decrease in neuroticism. In addition, Asendorpf and van Aken (2003) provided evidence that the quality of important social relationships, operationalized by perceived social support from peers, at the age of twelve influences personality characteristics five years later. The research mentioned so far primarily referred to the five-factor model or closely related constructs of personality. The present study is intended to extend previous findings and, therefore, aims to examine dynamic transactions between self-perceived social relationships and personality functioning in adolescents.

### 3.1 Personality functioning

**Figure 2:**
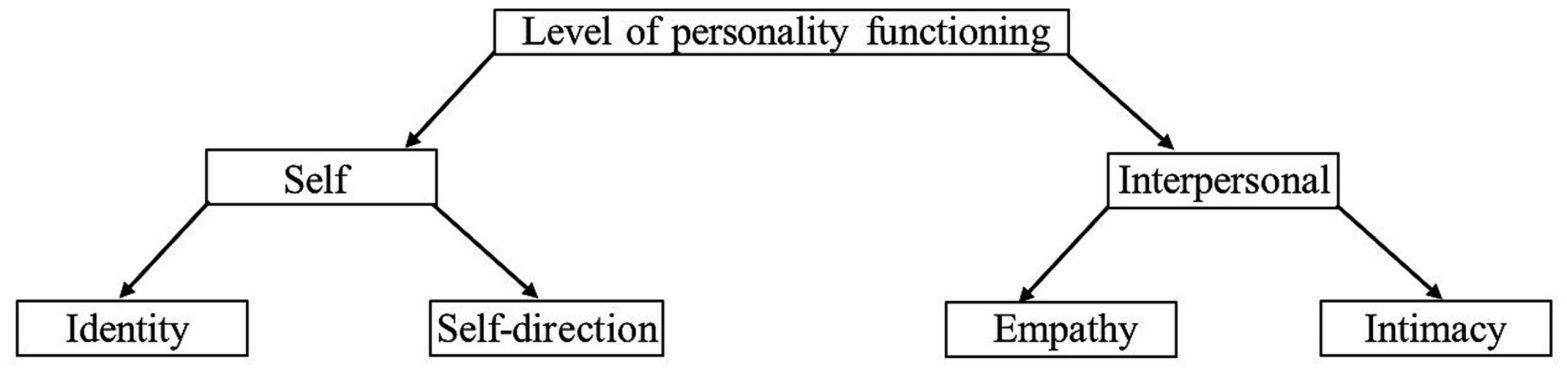
The global dimension, areas, and domains of personality functioning.

Adolescence is a sensitive phase of personality development, as a variety of physical, psychological, and social developmental tasks have to be mastered (Gniewosz and Titzmann, 2018). During this phase personality functioning and its development may become temporarily destabilized due to puberty (Neyer and Asendorpf, 2018). Therefore, adolescence is a critical and formative period in which fundamentals of psychosocial functioning are formed (Zimmermann et al., 2022). In adulthood, a reciprocal influence between personality and the perception and significance of social relationships is typically assumed. In this respect, these constructs are not independent of each other. However, in the normative development phase of puberty, relationship experiences appear to have a stronger influence on personality (Neyer et al., 2014). Thus, the perception of one’s relationship qualities in the classroom, which is determined by social-environmental factors, e.g., the support of classmates, have a stronger influence on personality characteristics than vice versa (Asendorpf and van Aken, 2003). This means that a positive perception of relationships in the classroom can lead to a stabilizing and healthy development of personality functioning. Goth et al. (2018a) have developed an age-specific inventory for adolescents which operationalize personality through the construct of personality functioning. It is an approach that combines developmental psychological aspects of adolescence with disorder-specific findings on personality pathologies (Goth et al., 2018a; Zimmermann et al., 2022). This concept was especially developed to take into account the developmental stage and life situation of adolescents and is intended to enable a differentiated diagnosis and therapy planning (*ibid*). Personality difficulties can originate in childhood and emerge in early adolescence (Dahl et al., 2018). Identification and treatment at an early stage is important, as adolescence is a critical and formative phase in which the foundations for psychosocial functioning are formed. This new approach enables a dimensional assessment of the level of personality functioning on a continuum from healthy personality development through personality difficulties to personality disorder and can, therefore, help in the diagnostic decision of adolescents (Zimmermann et al., 2022). Healthy personality functioning requires the development of self-capacities and interpersonal capacities. Therefore, the joint construct of personality functioning consists of two areas of functioning, namely the self and the interpersonal. The domains identity (me-integration) and self-direction (self-realization) form the area of the self. Empathy (prosocial social functioning) and intimacy (close personal relationships) form the interpersonal area (see Figure 2). The four domains are indicators of the global dimension of personality functioning and are based on the intra-and interpersonal functioning areas of personality described in the DSM-5 for dimensional description of the severity of personality disorders.

Studies (e.g., Hopwood et al., 2018) have already shown that the global dimension of personality functioning as well as partly the specific domains are related to the five-factors of personality (openness, conscientiousness, extraversion, agreeableness, and neuroticism) according to McCrae and Costa (1999). The strongest correlations were found between personality functioning and the factors neuroticism and agreeableness (Hopwood et al., 2018). In a validation study, small effects were found regarding gender differences for specific dimensions and differences between younger and older adolescents for the global dimension and the domains, except for empathy (Goth et al., 2018a).

### 3.2 Influencing factors on personality functioning: students’ self-perception of social relationships and special educational needs

School is a central place for building social relationships and personal competences (König et al., 2011; Rueger et al., 2016). In the current study, we deliberately focus on the students’ self-perception of their social relationships in inclusive classes, in order to capture the perspective of adolescents themselves. Bossaert et al. (2013) define students’ self-perception of their social relationships as all subjective impressions and feelings of students regarding their social situation within the classroom. As children grow older, their relationships with peers and their interpretations of relational experiences change, so that students’ self-perception of their social relationships and peer beliefs may play an increasingly larger role in the development of personality functioning (Ladd and Troop-Gordon, 2003).

Studies on students’ self-perception of their social relationships in inclusive learning settings have indicated that students with SEN perceive their peer relationships more negatively and feel less integrated in the social processes of their class compared to their peers without SEN (Koster et al., 2009; Schwab, 2015). This is particularly evident for students with SEN SED (Pijl et al., 2008; Crede et al., 2019; Hartmann et al., 2022). One explanation for this could be that the age-group appropriate social skills of students with SEN SED tend to be insufficiently developed (Pijl et al., 2008). Fonagy et al. (2015) suggested that behavioral deficits in childhood may lead to lower personality functioning in adolescence. Moreover, Ladd and Troop-Gordon (2003) assumed that both students’ behavioral or learning risks, such as aggression or developmental delays in school, and social relational risks, such as peer rejection or the feeling of not being integrated, increase the likelihood of mental dysfunctions. Therefore, the extent to which a students’ behavioral characteristic or special need develop in a healthy or maladjusted way depends on certain peer experiences (Gazelle and Ladd, 2003). Furthermore, Cambra and Silvestre (2003) found a positive relation between social acceptance (perception of classmates) and the development of personality self-concept (students’ self-evaluation of their personality) for students with SEN, but not for students without SEN. However, the association between the perspective of social relationships of students themselves and personality self-concept was not investigated. The aim of the present study is, therefore, to investigate whether the self-perception of students’ own social relationships in the classroom is related to or, moreover, impacts the self-perception of their personality functioning, e.g., self-regulation or ability to adopt perspectives.

## 4 Research questions and hypotheses

Overall, research provides evidence that negative social relationship experiences and SEN, especially in the domain of SED, can be considered as risk factors for the development of healthy personality functioning (Ladd and Troop-Gordon, 2003; Fonagy et al., 2015). To date, the association between adolescents’ self-perception of social relationships and their level of personality functioning and its development has not yet been investigated in the context of inclusive education, where students with and without SEN learn together. To close this research gap, a longitudinal study was conducted over the timespan of one school year with sixth- and seventh-graders in inclusive German schools. Both, students with and without SEN reported on their self-perception of social relationships within the classroom and their level of personality functioning. Based on the dynamic personality-relationship-transaction model (Neyer and Asendorpf, 2018), it is postulated that the relative strength and impact direction of transactions depends on the age group and context (Neyer et al., 2014; Asendorpf et al., 2017). Students of the present study were about twelve years old and, therefore, at the beginning of puberty (transition from childhood to adulthood). About half of the students were at the end of grade six and would transit from primary to secondary school in the following school year. The other half were in grade seven and had just made the transition. Both transitions are normative and should be accompanied by a stronger influence of social relationships on personality (Neyer et al., 2014). Hence, the following questions arise: How does students’ self-perception of their social relationships in class influence their level of personality functioning? Do students who report higher self-perception of their social relationships have healthier and, thus, less at-risk personality functioning? In particular, we hypothesize:

H1a: Students’ self-perception of their social relationships at the beginning of the school year (t1) is positively related to students’ personality functioning at the end of the school year (t2). This means that students who have a more positive self-perception of their social relationships at the beginning of the school year have healthier personality functioning at the end of the school year.

H1b: Students’ self-perception of their social relationships at t1 predicts positive changes in personality functioning over time: Students with higher self-perception of their social relationships have a stronger increase in healthy personality functioning over time compared to students with lower self-perception of their social relationships.

Studies have, moreover, indicated that children and adolescents with attention or behavior deficits were more likely to develop a personality disorder, i.e., lower personality functioning (Ladd and Troop-Gordon, 2003; Fonagy et al., 2015). Thus, the question arises if there are interindividual differences between students with and without SEN that influence the level of personality functioning and, further, lead to differential developmental trajectories in personality functioning. We assume that SEN L and SED could be seen as a possible risk factor in the development of personality functioning in adolescents. Therefore, we hypothesize:

H2a: SEN L at t1 is negatively related to students’ personality functioning at t2. This means that students with SEN L have a less healthy level of personality functioning than students without SEN.

H2b: SEN L at t1 predicts negative changes in students’ personality functioning over time: Students with SEN L have a decrease in healthy personality functioning over time compared to students without SEN.

H3a: SEN SED at t1 is negatively related to students’ personality functioning at t2, meaning that students with SEN SED have a less healthy level of personality functioning than students without SEN.

H3b: SEN SED at t1 predicts negative changes in students’ personality functioning over time: Students with SEN SED have a decrease in healthy personality functioning over time compared to students without SEN.

Gazelle and Ladd (2003) assumed that the extent to which a students’ behavioral characteristic or special need develop in a healthy or maladjusted way depends on certain peer experiences. Thus, it could be expected that the influence of self-perceived social relationships on personality functioning is stronger in students with SEN than in students without SEN. The question, therefore, arises whether students’ SEN moderates the influence of students’ self-perception of their social relationships on personality functioning. We hypothesize:

H4: SEN L reinforces the association of students’ self-perception of their social relationships at t1 and personality functioning at t2.

H5: SEN SED reinforces the association of students’ self-perception of their social relationships at t1 and personality functioning at t2.

Thus, H4 and H5 assume that for students with SEN L or SED, the relations between lower social self-perception and riskier levels of personality functioning is stronger.

With regard to the four domains of personality functioning (identity, self-direction, empathy, and intimacy), we generally assume similar patterns of impact.

## 5 Materials and methods

### 5.1 Sample and design

The present study is part of a longitudinal project on inclusive education in the federal state of Brandenburg in Germany, conducted between 2018–2020 (Lenkeit et al., 2023). The goal of the research project was to identify individual and contextual variables that influence the social and academic development of students with and without SEN in inclusive primary and secondary schools. Schools dedicated to being inclusive committed to include all students regardless of their SEN status. Instead of attending a special school, students with diagnosed or suspected SEN could learn in an inclusive mainstream school. In this inclusive learning environment, students with potential SEN are only formally diagnosed at the request of their parents. Thus, even when teachers suspect a SEN it does not necessarily lead to a formal diagnosis.

At the beginning of the study, we selected 52 schools using a stratified random sample from a total population of 184 project schools. For the present study we focused on adolescents. Consequently, we drew on information from about *N* = 927 students in 20 sixth- and 20 seventh-grade classes. In the federal state of Brandenburg, primary school comprises grade one to six, secondary school starts with grade seven. Our sample consisted of 449 (48.4%) sixth-graders of primary schools and 478 (51.6%) seventh-graders of comprehensive schools. Sixth-graders were on average 11.8 years (SD = 0.56) old of which 218 identified as female (48.6%) and 222 (49.4%) as male. Seventh-graders were on average 13 years (SD = 0.61) old with 213 female (44.6%) and 258 (54.0%) male students. Information on gender was missing for 16 students (1.7%). As psychometric diagnoses were non-obligatory, classroom teachers provided information whether a student had a formally diagnosed or suspected SEN in the one or more of the following domains: learning, social–emotional, language, sight, hearing, intellectual as well as motor skills and physical development (see Table 1). In grade six 68 (15.1%) students had a suspected or formally diagnosed SEN in one of the domains, 31 (6.9%) had SEN L and 27 (6.0%) SEN SED. In grade seven 130 (27.2%) students had a suspected or formally diagnosed SEN in one of the domains, 52 (10.9%) had SEN L and 61 (12.8%) SEN SED. Twelve (1.3%) students were suspected or diagnosed with both SEN L and SED.

**Table 1:** Distribution of the special educational needs mentioned.

	SEN learning	SEN SED	SEN language	SEN sight	SEN hearing	SEN ID	SEN PD
Sixth-graders (*N* = 449)	31	27	9	2	3	6	3
Seventh-graders (*N* = 478)	52	61	22	2	3	7	10

SED = Social–emotional development, PD = Physical and motor skills development, ID = Intellectual development. Multiple answers were possible.

### 5.2 Data collection

The data was collected in school year 2018/2019, in November 2018 (t1) and June 2019 (t2) in whole-class arrangements by trained test administrators. For students with SEN, alterations were administered when needed (additional time, personal assistant, or larger prints). The survey instruments and data collection were approved by the data protection officer of the Ministry of Education. Due to the project was issued by the Ministry of Education, participation was mandatory for classes and teachers. However, students’ participation was voluntary and they could either opt out of taking part at all or stop answering the questionnaire at any time. Parents were informed about the context and content of the survey. They were also informed that their children could stop participating in the survey process at any time.

### 5.3 Instruments

#### 5.3.1 Demographic data

We asked teachers at t1 to indicate students’ date of birth and gender, recoded into 0 for girls and 1 for boys. Parents information on their educational and occupational levels were used to calculate the international socio-economic index of occupational status (ISEI, Ganzeboom, 2010). The highest of both parents’ ISEI was used in the analysis (HISEI) to indicate the socioeconomic background of the family. In our sample the average HISEI was 48.89 (SD = 19.47). HISEI scores typically range from 16 (lower socio-economic background) to 90 (high socio-economic background).

#### 5.3.2 Special educational need

We obtained information on students’ SEN status through classroom teachers who indicated students with a formally or suspected SEN diagnosis. For each domain, answers were coded with 0 (no SEN) or 1 (SEN). As for schools investigated psychometric diagnoses were not mandatory, we have grouped students with formally diagnosed and suspected SEN together in order to include all students with SEN and not miss anyone. While Table 1 includes the number of students with formally diagnosed or suspected SEN in all domains, the Ministry of Education of the federal state of Brandenburg focuses on the inclusion of students with SEN in the domains of learning, social–emotional development, and language (Lange et al., 2016). This is seen as a first important step towards an inclusive system. The focus falls on these three domains, not only because (taken together) they comprise the largest number of students with SEN [KMK (Conference of the Ministers of Education and Cultural Affairs), 2022], and are interrelated with regard to their accompanying family background characteristics and support needs (Preuss-Lausitz, 2011). But also because, compared to other SEN domains related for example to hearing or visual impairments, it may be assumed that they are easier to integrate in regular schools with regularly educated teachers and, thus, seem to not immediately require substantial changes in teacher education (Lenkeit et al., 2022). Table 1 shows that students with SEN L and SED make up the largest proportion and that there are only few students with SEN language, particularly in grade six. Therefore, for the present analyses, students with SEN L and SED are considered. Students with SEN L are characterized by showing patterns of thinking and learning that can lead to disorientation when encountering and dealing with school learning objects. Students with SEN SED are characterized by difficulties in emotional and social development, cooperation, and self-regulation [KMK (Conference of the Ministers of Education and Cultural Affairs), 2022].

#### 5.3.3 Students’ self-perception of their social relationships

Students’ social self-perception within their class was measured at both measurement points on a four-point Likert scale consisting of six items adapted from Rauer and Schuck (2003). Ratings ranged from 0 (strongly disagree) to 3 (strongly agree). This established scale has already been used in previous studies in inclusive school contexts. With the following six items students indicated to which degree they perceive themselves as socially integrated within the class: “Only few classmates like me (−); My classmates are kind to me; Others listen when I talk; In our class, I feel comfortable; Others often laugh at me (−); I only have few friends in my class (−).” The internal consistency of the scale was good (t1: Cronbach’s α = 0.81; t2: Cronbach’s α = 0.84).

#### 5.3.4 Personality functioning

In order to operationalize personality functioning, the Level of Personality Functioning Questionnaire Screening version (LoPF-Q Screener; Zimmermann et al., 2022) was used. This self-report instrument is a short version with 20 Items derived from the original LoPF-Q 12–18 with 97 items (Goth et al., 2018b) aiming to provide high clinical validity and a sound latent factor structure. Both versions are designed to assess personality functioning in adolescence through self-reporting according to the DSM5 AMPD and the ICD-11 in order to support early detection of personality difficulties or disorder. The short screening version was developed to meet the need of efficient screening for personality difficulty in clinical and research applications. The LoPF-Q Screener provides a total score and four subscale scores representing difficulties in the functional domains of identity, self-direction, empathy, and capacity for intimacy. Each domain contains five items on a five-point Likert scale ranging from 0 (no), 1 (rather not), 2 (part/part), 3 (rather yes) to 4 (yes). At both measurement points, students were asked to indicate how much statements such as “I am confused myself, about what kind of person I truly am.” (identity), “I often suppress my desires.” (self-direction), “I am offended very quickly.” (empathy) or “I feel uncomfortable being asked about my feelings.” (intimacy) apply to them. The internal consistency for our sample was good (t1: total score α = 0.89; t2: total score α = 0.90). In the current study in an inclusive school context, high scores of “impaired personality functioning” in the LoPF-Q Screener are interpreted as a potential risk in personality functioning development while low scores are interpreted as a healthy status of personality functioning. Since the LoPF-Q Screener is just a short screening instrument and a self-report questionnaire, further diagnostics would be needed to clarify whether a personality disorder is present.

### 5.4 Statistical procedure

All analyses were performed using SPSS (Version 28, IBM Corp., 2021) and R (Version 4.1.3, R Core Team, 2022) software packages. Students were clustered in classes and we accounted for the resulting non-independence of observations drawing on multilevel modelling techniques (Raudenbush and Bryk, 2002). Analyses of intraclass correlations of personality functioning indicated dependencies in data classes (ICC grade six = 0.06; grade seven = 0.04). We calculated longitudinal two-level hierarchical regression analyses (R packages “nlme,” “lme4”, and “lmerTest”). This enabled us to distinguish between within-class and between-class associations and, furthermore, between within-student and between-student variance in terms of changes in personality functioning. Since higher LoPF scores indicate a riskier personality development, a decrease in LoPF scores implies an improvement in personality functioning. For all analyses, we used the aggregated means of the total raw score and the four domains of at-risk personality functioning. Prior to analyses, level 1 predictors were group-mean centered. Since sixth-graders in primary schools usually have known each other for a longer time and attended a different type of school than seventh-graders in secondary comprehensive schools, we performed all analyses separately for grades six and seven. In a first step (model 1) the total score of at-risk personality functioning was regressed on time (representing the change from t1 to t2), students’ self-perception of their social relationships, and the interaction of time and self-perception of social relationships. Additionally, we treated students’ age, gender, and HISEI as control variables. Next, at-risk personality functioning was separately regressed on SEN L (model 2a) and SEN SED (model 2b), and the interaction between time and SEN L, respectively, SED. In the last model 3, moderations between students’ self-perception of relationships and SEN L and SED were examined. Subsequently, we applied this modelling strategy separately for the four personality functioning domains: at-risk identity, self-direction, empathy, and at-risk capacity for intimacy as dependent variables, again, separately for grades six and seven. The longitudinal multilevel regression analyses were conducted in R using the full maximum likelihood estimator (FML). For all analyses, alpha was set at *p* < 0.05. Due to the absence of individual students, missing data occurred. The missing rate for self-perception of social relationships was 14.35% at t1 and 16.29% at t2. The missing rate for personality functioning was 14.35% at t1 and 17.26% at t2. We used multiple imputation to replace missing values (R package “mice”).

## 6 Results

### 6.1 Descriptive summary of at-risk personality functioning

Table 2 provides an overview of the means (M) and standard deviations (SD) for all students as well as for the subgroups of students with SEN L and SED, separately for grades six and seven. In both grades, students with SEN SED had higher at-risk personality functioning compared to all students and those with SEN L. Sixth-graders with SEN SED had higher LoPF scores than seventh-graders with SEN SED, implying a higher level of at-risk personality functioning. With regard to all students and those with SEN L seventh-graders had higher LoPF scores than sixth-graders.

Table 3 shows the correlations between self-perception of social relationships and the LoPF total score as well as with the four domains. In both grades, significant negative correlations between self-perception of social relationships and at-risk personality functioning were found. However, the strength of correlations does not indicate a conceptual overlap of the constructs (see Table 3).

**Table 2:** Descriptive statistics of the total score and the four domains of at-risk personality functioning.

	Sixth-graders			
		All students (*N* = 449) M (SD)	SEN L (*N* = 31) M (SD)	SEN SED (*N* = 27) M (SD)
t1	Total score	1.26 (0.71)	1.41 (0.80)	1.82 (0.68)
	Difficulties with identity	1.13 (0.82)	1.34 (0.83)	1.63 (0.83)
	Difficulties with self-direction	1.18 (0.86)	1.37 (1.06)	1.79 (0.92)
	Difficulties with empathy	1.36 (0.75)	1.43 (0.77)	1.91 (0.74)
	Difficulty with intimacy	1.38 (0.85)	1.49 (1.01)	1.94 (0.81)
t2	Total score	1.25 (0.71)	1.35 (0.74)	1.66 (0.75)
	Difficulties with identity	1.09 (0.78)	1.16 (0.91)	1.43 (0.81)
	Difficulties with self-direction	1.15 (0.83)	1.25 (0.90)	1.59 (0.82)
	Difficulties with empathy	1.37 (0.78)	1.46 (0.80)	1.72 (0.88)
	Difficulties with intimacy	1.41 (0.87)	1.54 (0.89)	1.93 (0.89)

	Seventh-graders			
		All students (*N* = 478) M (SD)	SEN L (*N* = 52) M (SD)	SEN SED (*N* = 61) M (SD)
t1	Total score	1.34 (0.71)	1.48 (0.80)	1.63 (0.60)
	Difficulties with identity	1.21 (0.80)	1.40 (0.95)	1.37 (0.82)
	Difficulties with self-direction	1.30 (0.90)	1.47 (1.02)	1.59 (0.85)
	Difficulties with empathy	1.46 (0.75)	1.47 (0.85)	1.88 (0.67)
	Difficulties with intimacy	1.42 (0.83)	1.59 (0.88)	1.69 (0.67)
t2	Total score	1.39 (0.76)	1.42 (0.84)	1.49 (0.66)
	Difficulties with identity	1.27 (0.84)	1.32 (0.85)	1.34 (0.81)
	Difficulties with self-direction	1.33 (0.95)	1.24 (1.07)	1.41 (0.92)
	Difficulties with empathy	1.46 (0.78)	1.42 (0.91)	1.62 (0.67)
	Difficulties with intimacy	1.52 (0.89)	1.67 (0.95)	1.59 (0.65)

Ratings were made on a five-point Likert scale (0–4) and then aggregated to a mean score per scale. Higher scores indicate a lower level of personality functioning.

**Table 3:** Correlations for sixth-graders and seventh-graders.

	Sixth-graders					Seventh-graders				
Variable	1	2	3	4	5	1	2	3	4	5
1 Self-perception of social relationships										
2 LoPF total score	−0.39*					−0.26*				
3 Difficulties with identity	−0.38*	0.86*				−0.18*	0.87*			
4 Difficulties with self-direction	−0.32*	0.90*	0.74*			−0.22*	0.90*	0.76*		
5 Difficulties with empathy	−0.31*	0.84*	0.57*	0.68*		−0.27*	0.84*	0.60*	0.69*	
6 Difficulties with intimacy	−0.37*	0.90*	0.69*	0.74*	0.69*	−0.26*	0.88*	0.69*	0.70*	0.70*

* *p* < 0.05.

### 6.2 Results for predicting the total score of at-risk personality functioning

**Figure 3:**
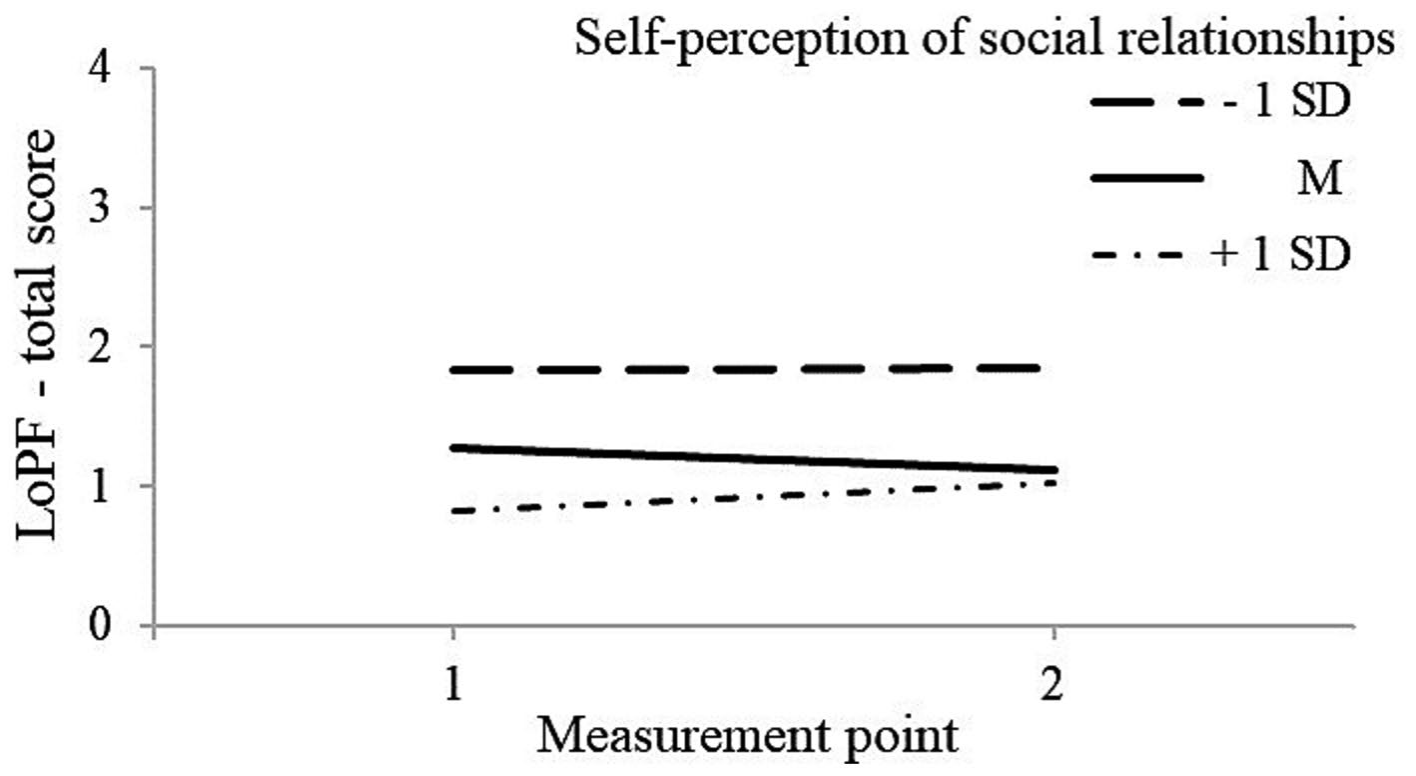
The influence of sixth-graders’ self-perception of social relationships on the change in at-risk personality functioning.

With regard to hypotheses 1a and 1b, in model 1 personality functioning was regressed on control variables, time, students’ self-perception of social relationships, and the interaction of time and social self-perception (see Tables 4, 5). For sixth-graders, we found no significant influence of the control variables (*p* > 0.05). But results indicate a significant negative influence of students’ self-perception of social relationships on at-risk personality functioning (*b* = −0.65, *p* < 0.001). That is, students who felt more integrated within the class had lower LoPF scores and, therefore, a higher level of personality functioning and less risky personality functioning. Additionally, we found a significant positive influence of students’ self-perception of social relationships on the change in personality functioning (*b* = 0.20, *p* = 0.001). Figure 3 shows that students with higher self-perception of their social relationships (+1 SD) had an increase of at-risk personality functioning (decrease of personality functioning) over time compared to students with lower self-perception of their social relationships (−1 SD). Note that the opposite direction of influence was assumed.

**Table 4:** Results for predicting the total score of at-risk personality functioning for sixth-graders.

	Model 0		Model 1		Modell 2a		Model 2b		Model 3	
	*b*	SE	*b*	SE	*b*	SE	*b*	SE	*b*	SE
Constant	1.26	0.03	1.21	0.04	1.17	0.04	1.19	0.04	1.10	0.05
*Control variables*										
Age			−0.04	0.06	−0.08	0.07	−0.07	0.07	−0.07	0.07
Gender			0.09	0.06	0.04	0.06	0.06	0.06	0.05	0.06
HISEI			−0.00	0.00	−0.00	0.00	−0.00	0.00	−0.00	0.00
*Predictors*										
Time			0.02	0.03	0.02	0.04	0.02	0.04	0.12*	0.05
Self-perception of social relationships			−0.65*	0.06	−0.71*	0.07	−0.64*	0.06	−0.75*	0.08
SEN L					0.07	0.15			−0.44	0.55
SEN SED							0.49*	0.16	−0.60	0.78
Self-perception of social relationships*Time			0.20*	0.06	0.27*	0.07	0.18*	0.06	0.26*	0.07
SEN L*Time					0.13	0.15			0.63	0.53
SEN SED*Time							−0.08	0.16	0.91	0.78
*Moderations*										
Self-perception of social relationships*SEN L									0.56	0.93
Self-perception of social relationships*SEN SED									−1.26	1.31
ICC (class level)	0.06									
ICC (student level)	0.52		0.54		0.53		0.52		0.53	
Explained inter-individual variance in %			6.28		14.35		15.25		17.49	
Explained intra-individual variance in %			66.86		67.61		67.42		69.32	

Longitudinal multilevel hierarchical regression analysis; Time = change from t1 to t2; *p < 0.05.

**Table 5:** Results for predicting the total score of at-risk personality functioning for seventh-graders.

	Model 0		Model 1		Modell 2a		Model2b		Model 3	
	*b*	SE	*b*	SE	*b*	SE	*b*	SE	*b*	SE
Constant	1.37	0.03	1.33	0.04	1.29	0.04	1.32	0.04	1.32	0.09
*Control* var*iables*										
Age			0.07	0.06	0.04	0.07	0.10	0.07	0.09	0.07
Gender			−0.21*	0.07	−0.24*	0.08	−0.29*	0.07	−0.28*	0.08
HISEI			−0.00	0.00	0.00	0.00	0.00	0.00	0.00	0.00
*Predictors*										
Time			0.02	0.04	0.03	0.04	0.01	0.04	−0.06	0.08
Self-perception of social relationships			−0.45*	0.07	−0.42*	0.08	−0.45*	0.08	−0.25	0.17
SEN L					0.21	0.14			−0.65	0.51
SEN SED							0.38*	0.13	0.84	0.50
Self-perception of social relationships*Time			0.06	0.07	−0.06	0.07	0.04	0.07	−0.03	0.08
SEN L*Time					−0.28*	0.12			−0.19	0.46
SEN SED*Time							−0.29*	0.12	−0.63	0.44
*Moderations*										
Self-perception of social relationships*SEN L									1.42	0.99
Self-perception of social relationships*SEN SED									0.26	0.97
ICC (class level)	0.04									
ICC (student level)	0.50		0.61		0.63		0.61		0.61	
Explained inter-individual variance in %			13.06		16.33		4.08		5.71	
Explained intra-individual variance in %			26.56		29.46		32.78		31.54	

Longitudinal multilevel hierarchical regression analysis; Time = change from t1 to t2; *p < 0.05.

For seventh-graders, model 1 shows a significant negative influence of the control variable gender (*b* = −0.21, *p* = 0.003). Thus, girls had significant higher LoPF scores than boys, implying a riskier personality functioning. Similar to sixth-graders, results indicate a significant negative influence of seventh-graders’ self-perception of social relationships on at-risk personality functioning (*b* = −0.45, *p* < 0.001). But we found no significant influence of students’ self-perception of social relationships on the change in personality functioning (*p* > 0.05).

For both grades, these findings support hypothesis 1a, but not hypothesis 1b because for seventh-graders we found no change over time in at-risk personality functioning and in grade six we found the opposite direction of change than assumed.

To test hypotheses 2a and 2b, in model 2a the level of personality functioning was additionally regressed on students’ SEN L. For both grades, SEN L had no significant effect on personality functioning (*p* < 0.05). But for seventh-graders SEN L had a negative influence on the change in personality functioning (*b* = −0.28, *p* = 0.025). This negative interaction indicates that students with SEN L showed a decrease in the LoPF scores over time (increased level of personality functioning and less risky personality development). While for students without SEN, LoPF scores increased. Thus, the differences between the two groups decreased over time. Therefore, hypotheses 2a and 2b have to be rejected as the opposite direction of change was hypothesized.

With regard to hypotheses 3a and 3b, in model 2b the level of personality functioning was regressed on SEN SED. For both grades, we found a significant positive influence of SEN SED on at-risk personality functioning (*b* = 0.49, *p* = 0.002; *b* = 0.38, *p* = 0.003). That is, students with SEN SED reported significantly higher LoPF scores (lower level of personality functioning) than their classmates without SEN. Additionally, in grade seven results indicate a significant negative influence of SEN SED on the change in personality functioning over time (*b* = −0.29, *p* = 0.014), which indicates that students with SEN SED showed a decrease in LoPF scores (increased level of personality functioning and less risky personality development) over time compared to students without SEN who showed an increase in LoPF scores. Thus, the differences between the two groups decreased over time. Hence, hypothesis 3a can be supported, but not hypothesis 3b because for sixth-graders we found no change over time, and for seventh-graders we found the opposite direction of change than was assumed.

In a last step (model 3) to test hypotheses 4 and 5, moderations between students’ self-perception of their social relationships and SEN L and SED were additionally examined. Therefore, personality functioning was regressed on the interactions of students’ self-perception of their social relationships and students’ SEN. For both grades, there was no significant moderation effect neither for students’ self-perception of their social relationships and SEN L (*p* > 0.05) nor for students’ self-perception of their social relationships and SEN SED (*p* > 0.05). SEN L and SED did not strengthen the influence of students’ self-perception of their social relationships on personality functioning. Therefore, hypotheses 4 and 5 are rejected. Remarkably, in grade six, this model revealed a significant positive change of time (*b* = 0.12, *p* = 0.014), indicating a decreased level of healthy personality functioning. SEN SED no longer appeared to have a significant influence (*p* > 0.05). But the negative influence of students’ self-perception of social relationships on at-risk personality functioning and the positive influence of students’ self-perception of social relationships on the change in at-risk personality functioning over time remained stable. This result once again supports hypothesis 1a for sixth-graders. In grade seven, however, students’ self-perception of social relationships no longer appeared to have a significant influence (*p* > 0.05). Only gender was found to have a significant influence on personality functioning: Girls had a significant lower level of personality functioning and, therefore, a riskier personality functioning than boys.

### 6.3 Results for predicting domains of at-risk personality functioning

To take a detailed look at the four different domains of at-risk personality functioning, the same sequence of models (model 0–3) was run for difficulties with identity, self-direction, empathy, and intimacy, again separately for grade six and seven. Overall, analyses for the four specific domains revealed similar patterns of findings to the total LoPF score.

In both grades analyses revealed a significant negative influence of students’ self-perception of social relationships on all four domains of personality functioning. These results once again support hypothesis 1a. As for the total score, we found no significant influence of seventh-graders’ self-perception of social relationships on changes in the four domains. In contrast, there was a significant positive influence of sixth-graders’ self-perception of social relationships on the change in identity, self-direction, empathy, and intimacy. However, the opposite direction of change was expected, which again contradicts hypothesis 1b.

As it was shown for the total score, SEN L had no significant effect on the domains of at-risk personality functioning in both grades, which supports the rejection of hypothesis 2a. In contrast to the total score, but in line with hypothesis 2b, results of sixth-graders indicate a significant positive influence of SEN L on the change in self-direction over time (*b* = 1.40, *p* = 0.047). This positive interaction means that six-graders with SEN L showed a riskier development of self-direction compared to students without SEN. This result supports hypothesis 2b. However, for seventh-graders, SEN L had a negative influence on the change in identity (*b* = −0.35, *p* = 0.026) and self-direction (*b* = −0.46, *p* = 0.007). This indicates that seventh-graders with SEN L showed a decrease in difficulties with identity and self-direction (increased level of healthy identity and self-direction) compared to students without SEN. This is in line with the total score, but contrary to hypothesis 2b.

Also in line with the total score, students of both grades with SEN SED reported significantly higher difficulties in all four domains and, thus, lower levels of healthy identity, self-direction, empathy, and intimacy compared to students without SEN. These results once again support hypothesis 3a. For seventh-graders, results indicate a significant negative influence of SEN SED on the change in identity, self-direction, and empathy over time. These negative interactions indicate that students with SEN SED showed a less risky development of identity, self-direction, and empathy compared to students without SEN. Again, these results oppose hypothesis 3b. For sixth-graders, hypothesis 3b is also rejected, as we did not find any effect of SEN on change in any of the domains.

For both grades, we found no significant moderation effects on difficulties in the domains of personality functioning, neither for students’ self-perception of their social relationships with SEN L nor with SEN SED. In line with the total score, hypotheses 4 and 5 have to be rejected.

## 7 Discussion

### 7.1 Summary of results

The aim of the present study was to investigate how students’ self-perception of their social relationships in class affects the development of personality functioning in adolescence. Furthermore, we examined whether special educational needs in the domains of learning (SEN L) and social–emotional development (SED) led to differential developmental trajectories of personality functioning compared to students without SEN. Finally, we investigated whether students’ SEN moderates the influence of students’ self-perception of their social relationships on personality functioning.

Overall seventh-graders who had recently made the transition from primary to secondary school showed higher at-risk personality functioning than sixth-graders. Moreover, in grade seven, students’ gender was found to be a significant predictor influencing the level of personality functioning. Compared to boys, girls had a lower level of healthy personality functioning. This was reflected in higher scores in at-risk personality functioning in the total score and the domains self-direction, identity, and intimacy.

With regard to the first research question, analyses of sixth- and seventh-graders revealed that students’ self-perception of their social relationships predicts the total score of personality functioning and, moreover, the four domains identity, self-direction, empathy, and intimacy. We can, thus state, that students who perceived their social relationships within the class more positively had a healthier perception of their own personality functioning (lower at-risk personality functioning). Therefore, our findings support the dynamic personality-relationship-transaction model by Neyer and Asendorpf (2018). Furthermore, results are also in line with findings from Ladd and Troop-Gordon (2003) who indicated that peer difficulties were associated with psychological dysfunction. Neyer et al. (2014) postulated a stronger influence of social relationships on personality in the context of normative life transitions. For sixth-graders, we were able to observe this direction of impact over all models. In grade seven, however, in the final model students’ self-perception of social relationships no longer appeared to be significant. One explanation for the more robust influence of social self-perception on personality functioning in grade six compared to grade seven may be the stage of the normative transition process. Seventh-graders have already made this normative transition from primary to secondary school and may have stored it away mentally at the time of survey. For sixth-graders, on the other hand, this normative transition from primary to secondary school was imminent and, thus, they seem to be more engaged in this transition process. Furthermore, the higher explained within-student variance among sixth-graders compared to seventh-graders (see Tables 4, 5) also supports that the dynamic personality-relationship-transaction model is more valid in grade six. However, we could not found the assumed changes over time. Hypothesis 1b stated that students with higher self-perception of their social relationships would show greater increases in healthy personality functioning over time than students with lower self-perceptions of their social relationships. In grade seven, the influence of self-perceived social relationships on the change in personality functioning and the four domains was not evident. In grade six, changes in personality functioning and the four domains were predicted by students’ self-perception of their social relationships. Contrary to hypothesis 1b, we found the opposite direction of change than assumed: Students with higher self-perception of their social relationships showed a slight increase in at-risk personality functioning compared to students with lower self-perception of social relationships. Consequently, a more positive self-perception of social relationships does not necessarily facilitate the development of healthier personality functioning.

In terms of the second research question, in both grades analyses revealed that students with SEN SED had a significantly lower level of healthy personality functioning as well as identity, self-direction, empathy, and intimacy. These findings are in line with prior research by Ladd and Troop-Gordon (2003). They found that children’s behavioral dispositions, especially aggressiveness, were related with mental functioning problems. These patterns of findings can be explained by the phenotype of SEN SED: these students are characterized by difficulties in goal resistance (self-direction), cooperation, social and emotional development [KMK (Conference of the Ministers of Education and Cultural Affairs), 2022] and, thus, are more likely to exhibit relatively higher levels of aggressive and unsocial behaviors. It should be noted that SEN SED comprises a heterogeneous spectrum of students who, however, often stand out negatively in their class. Teachers especially feel challenged by teaching students with SEN SED, as their behavioral difficulties may disrupt instructional and learning processes in the classroom (Blumenthal et al., 2020). Peers, too, often express more negative attitudes towards those with behavioral difficulties compared to those with learning difficulties (de Boer et al., 2012; Tsakiridou and Polyzopoulou, 2019). Inclusive schools, however, should offer children and adolescents social participation and equal opportunities regardless of their individual learning requirements. Ideally, the heterogeneity of a class is not only perceived and conceded to the students, but also seen as an opportunity for the didactic design of lessons. For a successful inclusion of all students, teacher education in Germany will have to emphasize preparing students teachers for the management of many diverse needs and academic prerequisites more than is currently done. Studies have already shown that effective classroom management has a positive impact on students’ social participation in inclusive classrooms (e.g., Garrote et al., 2020; Gasser et al., 2022). This is because classroom management routines can prevent disruptive behavior (Kostewicz et al., 2008) and, thus, prevent a negative perception of students with behavioral difficulties, which in turn can have a positive effect on the development of personality functioning. In addition, teachers’ responsibility to strengthen the social skills of all students is also important to help them recognize, respect, and interact with students who have a diverse set of strengths and difficulties (Bosse and Spörer, 2022). The analyses for sixth- and seven-graders with SEN L revealed no significantly lower level of healthy personality functioning or identity, self-direction, empathy, or intimacy. This could be due to the fact that the difficulties of students with SEN L relate more to academic aspects and less to social or behavioral difficulties as with students with SEN SED. Furthermore, SEN SED was found to be a significant predictor of changes in seventh-graders’ total score and identity, self-direction, and empathy. However, contrary to our hypothesis 3b, results indicate that seventh-graders with SEN SED had a positive development of healthy personality functioning as well as their identity, self-direction, and empathy compared to their classmates without SEN. These patterns of findings were also observed for seventh-graders with SEN L. Again, contrary to our hypothesis 2b, seventh-graders with SEN L had a positive development of personality functioning as well as their identity and self-direction. Therefore, these finding patterns are encouraging and indicate that in grade seven differences in personality functioning between students with and without SEN decreased over time. This could be due to the fact that students with SEN have been able to adapt well to their new comprehensive school. So, that their personality functioning has developed positively in the course of grade seven. Possibly because their SEN may no longer be as prominent as in primary school, where the differences between students may more pronounced. Thus, primary school classes are likely to be more heterogeneous compared to classes in comprehensive schools. In addition, inclusive comprehensive schools in particular may have found successful ways to support students with special needs to adapt well to the new situation. This allows students with SEN to develop more positively in their personal functioning.

Contrary to our last hypotheses, we found no significant moderation effects, neither in grade six nor in grade seven. It was assumed that personal characteristics, such as developmental difficulties in school, and their social relationships have combined effects on personality development as previous studies have shown (e.g., Cambra and Silvestre, 2003; Ladd and Troop-Gordon, 2003). But personality functioning was not predicted by the interaction of students’ self-perception of their social relationships and SEN. Neither SEN L nor SEN SED does strengthen the influence of students’ self-perception of their social relationships in class on personality functioning or on identity, self-direction, empathy, and intimacy.

Nevertheless, our study extends previous research on personality functioning in adolescents by focusing on the influence of students’ self-perception of their social relationships on personality functioning in the context of primary and secondary inclusive educational settings.

### 7.2 Limitations and future prospects

This study has, however, limitations that should not go unnoticed. A first constraint relates to the measurement of students’ SEN. Classroom teachers were asked to provide information whether a student had a formally diagnosed or suspected SEN. We have no information about the psychometric quality of teachers’ assessments of suspected SEN. Moreover, by combining formally diagnosed and suspected SEN, the proportion slightly exceeds the prevalence of SEN diagnoses in the German population [KMK (Conference of the Ministers of Education and Cultural Affairs), 2022]. Since inclusive learning includes the ability to avoid diagnoses as part of the framework, as for schools investigated, we have grouped students with formally diagnosed and suspected SEN together. This was necessary in order to include all students with SEN and not miss anyone. Teachers should, thus, also assess whether students are suspected of having SEN. If we had not proceeded in this way, students who need permanent special support, e.g., in mastering learning tasks, but do not have an official diagnosis, would also have been in the group of students without SEN due to the voluntary nature of diagnoses in inclusive schools in Germany. Furthermore, teachers also play an important role in the diagnostic process for identifying SEN, as they often inform parents of children’s suspected SEN. Based on the teachers’ classroom observations, diagnostic procedures are carried out at the parents’ request. Future studies should consider how learning and social–emotional difficulties can be assessed not categorically, but possibly through continuous rating procedures.

A second constraint relates to the assessment of social relationships. In the present study, students’ self-perception of their social relationships within the class was assessed. Thus, social relationships with other adolescents outside the class were not considered. In adolescence, other peers are certainly relevant for personality development, for instance friends from sports clubs or neighborhood. For example, Wagner et al. (2015) showed that the first partnership has the potential to influence personality and its development, whereby the partner may not be from the student’s own class. Future studies should consider enhancing the measurement by including the self-perception of social relationships outside the classroom or school. Moreover, it might be useful to add a peer-rated measure of social relationships, e.g., received friendship choices. In this way, the subjective social self-perception of adolescents could be expanded to take into account the external assessments by classmates.

Third, the LoPF-Q Screener is an instrument for detecting clinically relevant difficulties in personality functioning in order to inform diagnostic decision making towards personality disorders at an early stage of development in adolescents. However, the LoPF-Q is merely a screening instrument for self-report and additional diagnostics including different sources would have been needed to clarify whether a personality disorder was present or not. But this was not part of the research project and the identification of students who showed elevated scores in at-risk personality functioning would have violated the project’s anonymization policy. Nevertheless, the LoPF-Q Screener can be useful for assessing personality functioning that are relevant in the developmental period of adolescence.

And finally, the last limitation relates our number of measurement points. Students’ ratings were assessed at the beginning and the end of one school year. Referring to the variable time, no significant changes in personality functioning (except model 3 for sixth-graders) had emerged during this short time span. A longer time interval between time points or the addition of more measurement points would be beneficial.

### 7.3 Implications and conclusion

The findings revealed that the total score as well as the domains of personality functioning primarily depend on students’ self-perception of their social relationships in class. Moreover, students with SEN SED showed lower levels of the personality functioning. Students with SEN SED, therefore, appear to require special support in order to achieve healthy personal development in inclusive education. However, differences between students with SEN and without SEN decreased over the course of grade seven, but not grade six. These results underline the importance of positively perceived social interactions of all students in an inclusive learning environment, especially in primary school. Otherwise, risky personality development may be a consequence. The results stress the need to foster positive classroom-based social processes. Supporting the social competences of all students as well as promoting prosocial behavior of students with SEN SED seems to be an essential but challenging task for teachers. To promote their students’ social competences and prosocial behavior, one option might be to integrate a social skills training into their curricula (e.g., Garrote and Sermier Dessemontet, 2015). Furthermore, teachers should be trained to create an emotionally supportive and trust-based environment so that students do not have to be afraid to voice their problems.

Taken together, the need to belong to a peer group becomes increasingly important as students grow older as well as in the context of normative transitions (Rodkin and Ryan, 2012; Bossaert et al., 2013; Neyer et al., 2014). The reported findings suggest that positive perceived social relationships in class, in turn, have a significant influence on healthier development of personality functioning. While the presence of a SEN represents a potential risk factor for personality functioning, we observed that difference between seventh-graders with SEN L or SED and without SEN decreased over time. Furthermore, SEN does not appear to reinforce the association between low self-perception of social relationships and risky personality functioning. Consequently, our findings imply the chance that the influence of risk factors may be reduced when students feel well integrated within the classroom.

## Data availability statement

The raw data supporting the conclusions of this article will be made available by the authors, without undue reservation.

## Ethics statement

The study involving humans was approved by the Ethics Committee of the Ministry of Education, Youth, and Sports of the federal state of Brandenburg, Heinrich-Mann-Allee 107, 14473 Potsdam, Germany. The study was conducted in accordance with local legislation and institutional requirements. ‘Written informed consent for participation was not required from the participants or the participants’ legal guardians/next of kin in accordance with §1(2) of the Ordinance on the Approval of Scientific Studies in Schools of June 15, 2018, of the Brandenburg School Act, decreed by the Minister of Education, Youth and Sport.

## Author contributions

AH: Conceptualization, Data curation, Formal analysis, Investigation, Methodology, Project administration, Validation, Visualization, Writing – original draft, Writing – review & editing. MK: Conceptualization, Investigation, Project administration, Validation, Writing – review & editing. JL: Conceptualization, Investigation, Project administration, Validation, Writing – review & editing, Data curation. AE: Investigation, Project administration, Validation, Writing – review & editing. KG: Writing – review & editing. NS: Conceptualization, Funding acquisition, Investigation, Project administration, Resources, Supervision, Validation, Writing – review & editing.
