# Supplementary material for: The influence of adolescents’ self-perception of social relationships on personality functioning in the context of inclusive education
Source: Front Psychol. 2024 Aug 1;15:1279623. doi: 10.3389/fpsyg.2024.1279623 (PMC11324569; doi:10.3389/fpsyg.2024.1279623)
Supplement: Supplementary file 1 [file Table_1.pdf]

Table 1

Results for predicting difficulties with identity for sixth-graders

	Model 0		Model 1		Modell 2a		Model 2b		Model 3	
	b	*SE*	*b*	*SE*	*b*	*SE*	*b*	*SE*	*b*	*SE*
Constant	1.11	0.04	1.05	0.04	1.01	0.04	1.03	0.04	0.96	0.05
Control variables										
Age			-0.04	0.07	-0.08	0.07	-0.06	0.07	-0.07	0.08
Gender			0.02	0.07	-0.03	0.07	-0.01	0.07	-0.01	0.07
HISEI			-0.00	0.00	-0.00	0.00	-0.00	0.00	-0.00	0.00
Predictors										
Time			-0.01	0.04	-0.00	0.04	-0.01	0.04	0.07	0.05
Self-perception of social relationships			-0.62*	0.07	-0.66*	0.07	-0.63*	0.07	-0.70*	0.10
SEN L					0.16	0.18			0.06	0.63
SEN SED							0.47*	0.18	-0.75	0.89
Self-perception of social relationships*Time			0.18*	0.07	0.25*	0.07	0.18*	0.07	0.26*	0.07
SEN L*Time					0.01	0.17	-0.12	0.18	-0.40	0.60
SEN SED*Time									1.67	0.88
Moderations										
Self-perception of social relationships*SEN L									0.13	0.17
Self-perception of social relationships*SEN SED									0.22	0.16
ICC (class level)	0.08									
ICC (student level)	0.42									
Explained inter-individual variance in %			11.37		18.39		19.40		18.73	
Explained intra-individual variance in %			43.80		45.50		46.72		49.88	

Note: Longitudinal multilevel hierarchical regression analysis; Time = change from t1 to t2; *$p < .05$.

Table 2

Results for predicting difficulties with self-direction for sixth-graders

	Model 0		Model 1		Modell 2a		Model 2b		Model 3	
	b	*SE*	*b*	*SE*	*b*	*SE*	*b*	*SE*	*b*	*SE*
Constant	1.17	0.04	1.13	0.04	1.08	0.04	1.10	0.04	1.04	0.06
Control variables										
Age			0.00	0.08	-0.04	0.08	-0.02	0.08	-0.04	0.08
Gender			0.05	0.08	-0.02	0.08	-0.01	0.08	-0.03	0.08
HISEI			-0.00	0.00	-0.01*	0.00	-0.01*	0.00	-0.01*	0.00
Predictors										
Time			-0.01	0.05	-0.00	0.05	-0.01	0.05	0.10	0.06
Self-perception of social relationships			-0.68*	0.08	-0.75*	0.08	-0.67*	0.08	-0.76*	0.10
SEN L					0.14	0.19			-0.91	0.67
SEN SED							0.65*	0.20	0.61	0.95
Self-perception of social relationships*Time			0.24*	0.08	0.31*	0.09	0.20*	0.08	0.28*	0.09
SEN L*Time					0.10	0.20			1.40*	0.71
SEN SED*Time							-0.13	0.21	0.06	1.03
Moderations										
Self-perception of social relationships*SEN L									0.40	1.10
Self-perception of social relationships*SEN SED									-0.76	1.56
ICC (class level)	0.04									
ICC (student level)	0.46									
Explained inter-individual variance in %			12.26		21.70		24.84		27.67	
Explained intra-individual variance in %			18.50		19.84		20.11		24.13	

Note: Longitudinal multilevel hierarchical regression analysis; Time = change from t1 to t2; *$p < .05$.

Table 3

Results for predicting difficulties with empathy for sixth-graders

	Model 0		Model 1		Modell 2a		Model 2b		Model 3	
	b	*SE*	*b*	*SE*	*b*	*SE*	*b*	*SE*	*b*	*SE*
Constant	1.36	0.03	1.33	0.04	1.29	0.04	1.32	0.04	1.20	0.05
Control variables										
Age			-0.07	0.07	-0.09	0.07	-0.10	-0.07	-0.08	0.08
Gender			0.29*	0.07	0.24*	0.07	0.27*	0.07	0.25*	0.07
HISEI			-0.00	0.00	-0.00	0.00	0.00	0.00	-0.00	0.00
Predictors										
Time			0.02	0.04	0.04	0.04	0.01	0.04	0.12*	0.05
Self-perception of social relationships			-0.58*	0.07	-0.63*	0.07	-0.56*	0.07	-0.63*	0.10
SEN L					-0.08	0.17			-0.63	0.62
SEN SED							0.43*	0.18	-0.86	0.88
Self-perception of social relationships*Time			0.18*	0.07	0.25*	0.07	0.14*	0.07	0.23*	0.07
SEN L*Time					0.23	0.16			0.76	0.59
SEN SED*Time							-0.35	0.18	0.62	0.86
Moderations										
Self-perception of social relationships*SEN L									1.71	1.05
Self-perception of social relationships*SEN SED									-1.91	1.49
ICC (class level)	0.02									
ICC (student level)	0.44									
Explained inter-individual variance in %			2.41		0.40		2.01		2.41	
Explained intra-individual variance in %			29.72		34.67		33.75		38.39	

Note: Longitudinal multilevel hierarchical regression analysis; Time = change from t1 to t2; *p < .05.

Table 4

Results for predicting difficulties with intimacy for sixth-graders

	Model 0		Model 1		Modell 2a		Model 2b		Model 3	
	b	*SE*	*b*	*SE*	*b*	*SE*	*b*	*SE*	*b*	*SE*
Constant	1.40	0.04	1.33	0.04	1.29	0.04	1.31	0.04	1.18	0.06
Control variables										
Age			-0.07	0.08	-0.11	0.08	-0.11	0.08	-0.11	0.08
Gender			0.03	0.08	-0.05	0.08	0.01	0.08	-0.01	0.08
HISEI			-0.00	0.00	-0.00	0.00	-0.00	0.00	-0.00	0.00
Predictors										
Time			0.06	0.05	0.05	0.05	0.06	0.05	0.17*	0.07
Self-perception of social relationships			-0.74*	0.08	-0.79*	0.08	-0.72*	0.08	-0.90*	0.10
SEN L					0.08	0.19			-0.27	0.69
SEN SED							0.42*	0.20	-1.41	0.98
Self-perception of social relationships*Time			0.19*	0.09	0.27*	0.09	0.18*	0.09	0.27*	0.09
SEN L*Time					0.19	0.21			0.72	0.76
SEN SED*Time							0.27	0.22	1.37	1.11
Moderations										
Self-perception of social relationships*SEN L									-0.04	1.11
Self-perception of social relationships*SEN SED									-1.76	1.57
ICC (class level)	0.03									
ICC (student level)	0.40									
Explained inter-individual variance in %			17.13		24.83		23.08		26.92	
Explained intra-individual variance in %			20.05		19.36		22.78		24.15	

Note: Longitudinal multilevel hierarchical regression analysis; Time = change from t1 to t2; **p* < .05.

Table 5

Results for predicting difficulties with identity for seventh-graders

	Model 0		Model 1		Modell 2a		Model2b		Model 3	
	b	*SE*	*b*	*SE*	*b*	*SE*	*b*	*SE*	*b*	*SE*
Constant	1.24	0.04	1.18	0.04	1.17	0.05	1.16	0.05	1.24	0.09
Control variables										
Age			0.14*	0.07	0.10	0.08	0.10	0.07	0.09	0.08
Gender			-0.28*	0.08	-0.32*	0.08	-0.33*	0.08	-0.33*	0.09
HISEI			0.00	0.00	0.00	0.00	0.00	0.00	-0.00	0.00
Predictors										
Time			0.01	0.04	0.00	0.05	0.03	0.05	-0.14	0.10
Self-perception of social relationships			-0.46*	0.08	-0.43*	0.09	-0.44*	0.09	-0.24	0.18
SEN L					0.25	0.16			-0.90	0.56
SEN SED							0.24	0.15	1.43*	0.55
Self-perception of social relationships*Time			0.18*	0.08	0.05	0.09	0.14	0.09	0.02	0.10
SEN L*Time					-0.35*	0.16			0.17	0.57
SEN SED*Time							-0.14	0.15	-1.38*	0.55
Moderations										
Self-perception of social relationships*SEN L									1.12	1.06
Self-perception of social relationships*SEN SED									0.55	1.03
ICC (class level)	0.05									
ICC (student level)	0.34									
Explained inter-individual variance in %			9.73		8.56		4.67		0.39	
Explained intra-individual variance in %			46.78		47.76		48.93		49.90	

Note: Longitudinal multilevel hierarchical regression analysis; Time = change from t1 to t2; $*p < .05$.

Table 6

Results for predicting difficulties with self-direction for seventh-graders

	Model 0		Model 1		Modell 2a		Model2b		Model 3	
	b	*SE*	*b*	*SE*	*b*	*SE*	*b*	*SE*	*b*	*SE*
Constant	1.30	0.04	1.27	0.05	1.22	0.05	1.26	0.05	1.28	0.10
Control variables										
Age			0.19*	0.08	0.18*	0.09	0.26*	0.08	0.26*	0.09
Gender			-0.30*	0.09	-0.35*	0.09	-0.41*	0.09	-0.42*	0.10
HISEI			-0.00	0.00	-0.00	0.00	-0.00	0.00	-0.00	0.00
Predictors										
Time			0.01	0.05	0.04	0.05	0.02	0.05	-0.01	0.11
Self-perception of social relationships			-0.47*	0.09	-0.42*	0.10	-0.47*	0.10	-0.27	0.20
SEN L					0.26	0.18			-0.68	0.63
SEN SED							0.48*	0.16	1.09	0.61
Self-perception of social relationships*Time			0.03	0.09	-0.13	0.10	-0.02	0.10	-0.14	0.11
SEN L*Time					-0.46*	0.17			-0.16	0.63
SEN SED*Time							-0.42*	0.16	-0.54	0.61
Moderations										
Self-perception of social relationships*SEN L									1.86	1.19
Self-perception of social relationships*SEN SED									-0.37	1.16
ICC (class level)	0.04									
ICC (student level)	0.44									
Explained inter-individual variance in %			2.99		6.25		7.88		8.70	
Explained intra-individual variance in %			26.98		32.12		35.12		34.05	

Note: Longitudinal multilevel hierarchical regression analysis; Time = change from t1 to t2; *p < .05.

Table 7

Results for predicting difficulties with empathy for seventh-graders

	Model 0		Model 1		Modell 2a		Model2b		Model 3	
	b	*SE*	*b*	*SE*	*b*	*SE*	*b*	*SE*	*b*	*SE*
Constant	1.46	0.03	1.46	0.04	1.41	0.04	1.46	0.04	1.46	0.09
Control variables										
Age			-0.07	0.06	-0.10	0.07	-0.03	0.07	-0.02	0.08
Gender			-0.02	0.07	-0.04	0.08	-0.11	0.08	-0.08	0.08
HISEI			-0.00	0.00	0.00	0.00	0.00	0.00	0.00	0.00
Predictors										
Time			-0.04	0.04	-0.04	0.05	-0.07	0.05	-0.14	0.09
Self-perception of social relationships			-0.42*	0.08	-0.38*	0.09	-0.41*	0.09	-0.26	0.17
SEN L					0.16	0.15			-0.35	0.54
SEN SED							0.46*	0.14	0.69	0.53
Self-perception of social relationships*Time			0.04	0.08	-0.04	0.09	0.02	0.09	0.01	0.10
SEN L*Time					-0.19	0.15			-0.47	0.56
SEN SED*Time							-0.32*	0.15	-0.41	0.54
Moderations										
Self-perception of social relationships*SEN L									1.61	1.02
Self-perception of social relationships*SEN SED									-0.29	0.99
ICC (class level)	0.02									
ICC (student level)	0.37									
Explained inter-individual variance in %			14.22		19.91		5.21		9.95	
Explained intra-individual variance in %			23.96		27.58		30.08		31.20	

Note: Longitudinal multilevel hierarchical regression analysis; Time = change from t1 to t2; **p* < .05.

Table 8

Results for predicting difficulties with intimacy for seventh-graders

	Model 0		Model 1		Modell 2a		Model2b		Model 3	
	b	*SE*	*b*	*SE*	*b*	*SE*	*b*	*SE*	*b*	*SE*
Constant	1.47	0.03	1.41	0.05	1.36	0.05	1.38	0.05	1.29	0.11
Control variables										
Age			0.02	0.08	-0.03	0.09	0.05	0.08	0.05	0.09
Gender			-0.23*	0.08	-0.25*	0.09	-0.30*	0.09	-0.29*	0.10
HISEI			0.00	0.00	0.00	0.00	0.00	0.00	-0.00	0.00
Predictors										
Time			0.07	0.04	0.10*	0.05	0.07	0.05	0.07	0.10
Self-perception of social relationships			-0.46*	0.09	-0.46*	0.10	-0.47*	0.10	-0.18	0.20
SEN L					0.19	0.17			-0.66	0.63
SEN SED							0.33*	0.16	0.17	0.62
Self-perception of social relationships*Time			-0.02	0.08	-0.09	0.09	0.01	0.09	-0.03	0.10
SEN L*Time					-0.14	0.16			-0.20	0.57
SEN SED*Time							-0.24	0.15	-0.12	0.55
Moderations										
Self-perception of social relationships*SEN L									1.13	1.23
Self-perception of social relationships*SEN SED									1.26	1.20
ICC (class level)	0.03									
ICC (student level)	0.43									
Explained inter-individual variance in %			21.79		27.24		24.04		26.28	
Explained intra-individual variance in %			31.77		33.25		40.64		37.19	

Note: Longitudinal multilevel hierarchical regression analysis; Time = change from t1 to t2; *p < .05.
